# Echocardiographic Screening of Liver Transplant Candidates—Prevalence of Features of Portopulmonary Hypertension

**DOI:** 10.3390/jcm13226990

**Published:** 2024-11-20

**Authors:** Olga Dzikowska-Diduch, Tomasz Cader, Krzysztof Jankowski, Aisha Ou-Pokrzewińska, Monika Sznajder, Jan Siwiec, Szymon Pucyło, Aleksandra Sikora, Marek Pacholczyk, Wojciech Lisik, Piotr Pruszczyk, Katarzyna Kurnicka

**Affiliations:** 1 Department of Internal Medicine & Cardiology, Medical University of Warsaw, 02-091 Warsaw, Poland; olga.dzikowska-diduch@wum.edu.pl (O.D.-D.); aishaou@gmail.com (A.O.-P.); monikasznajder@o2.pl (M.S.); jjsiwiec@gmail.com (J.S.); spucylo@gmail.com (S.P.); sikora.alk@gmail.com (A.S.); piotr.pruszczyk@wum.edu.pl (P.P.); katarzyna.kurnicka@wum.edu.pl (K.K.); 2 Department of Social Medicine and Public Health, Medical University of Warsaw, 02-106 Warsaw, Poland; krzysztof.jankowski@wum.edu.pl; 3 Department of General and Transplant Surgery, Medical University of Warsaw, 02-091 Warsaw, Poland; marek.pacholczyk@wum.edu.pl (M.P.); wojciech.lisik@wum.edu.pl (W.L.)

**Keywords:** echocardiographic probability of pulmonary hypertension, portopulmonary hypertension, hepatopulmonary syndrome, liver transplantation

## Abstract

**Background**: The prevalence of portopulmonary hypertension (PoPH) is relatively low; however, its presence significantly worsens patients’ prognosis. When diagnosed, PoPH can be effectively treated, and specific therapies can lead to a substantial reduction in pulmonary circulation pressure, facilitating the safe performance of liver transplantation. Echocardiography is recommended as a first-line method for the non-invasive diagnosis of pulmonary hypertension and serves as a valuable screening tool for patients being evaluated for liver transplantation (LT). The objective of this study was to thoroughly assess the occurrence of echocardiographic signs indicative of pulmonary hypertension and hepatopulmonary syndrome (HPS) in candidates for LT. We assumed that our analysis also made it possible to assess how frequently these candidates require further invasive diagnostics for pulmonary hypertension at specialized centers and how often they may need targeted treatment for pulmonary arterioles as a bridge to transplantation, which could improve patient outcomes. Additionally, this study included a comprehensive review of the current literature. **Methods**: All LT candidates underwent standardized transthoracic echocardiography and contrast evaluation to identify intrapulmonary vascular shunts. **Results**: A total of 152 liver transplantation candidates (67 women, mean age 50.6 years) were included in the analysis. The estimated echocardiographic probability of pulmonary hypertension was classified as high in only one patient. However, 63 patients exhibited the visualization of microbubbles in the left heart chambers after an average of six cardiac cycles (ranging from three to nine cycles) following their appearance in the right heart. **Conclusions**: Our analysis shows that the features of PoPH and a high probability of PH were very rare in the LT candidates, and echocardiographic signs suggestive of hepatopulmonary syndrome were more prevalent. Liver transplant candidates need screening for PoPH and HPS, as both PoPH and HPS significantly worsen their prognosis, but specific PH treatment as a bridge to transplantation improves PoPH patients’ survival.

## 1. Introduction

Portopulmonary hypertension (PoPH) is classified as part of group 1 of pulmonary arterial hypertension (PAH) [1]. In comparison to other forms in group 1 of PAH, it is characterized by a better hemodynamic profile, but it has also been identified as having a higher risk cohort according to the US REVEAL [2] and Spanish REHAP [3] registry. In the American REVEAL registry, authors found that PoPH patients presented a higher average cardiac index, a lower right atrial pressure, and a higher 6 min walking distance (6 MWD) than average; however, they received less PAH-specific therapy than patients with idiopathic pulmonary arterial hypertension (IPAH) [2]. This is consistent with the conclusions of the Spanish REHAP registry, which is suggestive of PoPH patients being undertreated and showing poorer survival [3]. Moreover, portal hypertension was identified as a risk factor for death among patients included in the French PAH registry [4]. Liver disease-related events, in addition to inadequate treatment, have a major impact on the outcome and survival of patients with PoPH [4].

According to the registers, the prevalence of PoPH is relatively low. There is an estimate that the incidence of pulmonary arterial hypertension associated with portal hypertension is one case in every three million cases per year [1,5]. However, in the French study, PoPH was the third most common form of PAH [4]. In the past, it was thought that portal hypertension caused 5–10% of PAH cases, but with the widespread use of screening tests, this has risen to 15% of PAH patients [5]. One to ten percent of patients with portal hypertension develop pulmonary arterial hypertension [6]. Liver disease does not have to be an underlying condition of portal hypertension; however, in the vast majority of cases, portal hypertension is the result of a cirrhosis of the liver. Liver fibrosis causes an imbalance between constrictors and vasodilators, as well as increased flow through pulmonary circulation. This leads to endothelial injury and the dysfunction of pulmonary artery endothelial cells [6]. Additionally, systemic toxins and inflammation cause the proliferation of endothelial cells, the hypertrophy of smooth muscles, and in situ thrombosis.

PoPH worsens the prognosis of PAH candidates and is a significant cause of mortality. However, if PH is diagnosed, it can be effectively treated. Therefore, screening is recommended, and patients should be referred more frequently to centres that provide detailed PH diagnostics and have the possibility of commencing their specific management.

Screening, including an annual electrocardiogram, echocardiography, and BNP testing, should be considered for candidates for transplantation even in the absence of symptoms [5].

If a patient is a candidate for liver transplantation (LT), POPH screening should be repeated during the wait-list period.

As per the guidelines of the European Society of Cardiology (ESC), transthoracic echocardiography is the first non-invasive way to evaluate patients with suspected PH [1,5]. Moreover, the American Association for the Study of Liver Diseases recommends Doppler echocardiography for all LT patients [6].

The diagnosis of pulmonary hypertension can only be made on the basis of invasive measurements in the pulmonary arteries. Right heart catheterization (RHC) is not suitable for all patients. Currently, there is less consensus regarding the cut-off criteria for RHC based on echocardiography. PoPH should be confirmed by RHC to ensure its presence whenever the estimated right ventricular systolic pressure (eRVSP) of a LT candidate is greater than 50 mmHG [6] and, according to Korbitz, if the eRVSP is ≥45 mmHg [7]. As recommended by Raevens et al., LT candidates with an eRVSP > 38 mmHg ought to be referred for RHC, since a value of 38 mmHg has a maximum specificity of 82% and, at the same time, a sensitivity and negative predictive value of 100% [8]. RHC has been recommended for patients whose eRVSP exceeds 45 mmHg, according to the American Association for the Study of Liver Diseases. However, the International Liver Transplant Society recommends RHC for patients whose eRVSP exceeds 50 mmHg and/or who exhibit symptoms of right ventricular hypertrophy or malfunction [6].

According to the latest ESC guidelines, pulmonary arterial hypertension is defined as a mean arterial pressure over 20 mmHg, a pulmonary vascular resistance (PVR) over 2 Wood units’, and wedging pressure of under 15 mmHg. Compared to patients with IPAH, pulmonary hypertension associated with portal hypertension usually has a higher cardiac index and a lower PVR [5]. A diagnosis of portal hypertension during RHC can be made by determining if there is a significant gradient between the pressure measured in the hepatic veins before and after wedging. Moreover, an elevated wedge hepatic vein pressure and a free hepatic vein pressure over 5 mmHg indicate portal hypertension. Pulmonary portal hypertension is diagnosed after excluding other conditions that may be responsible for it, such as hyperkinetic circulation, volume overload, left ventricular diastolic dysfunction, lung diseases, and obstructive sleep apnoea.

As a result of the high mortality rate in pulmonary hypertension patients undergoing LT, even patients with an intermediate probability of it should be referred to RHC, which presents no significant risks. When patients with pulmonary hypertension are not diagnosed and left untreated, their prognosis worsens significantly, so it is preferable to perform an “unnecessary” RHC than to miss out on a diagnosis.

It is noteworthy that mortality rates have decreased with the use of pulmonary vasodilators and successful reductions in pulmonary arterial pressure in patients with a mPAP > 35 mmHg. Therefore, early detection may improve patients’ prognosis and survival [5,6].

Krowka et al. divided patients with PoPH based on their PH severity into three groups: those with mild pulmonary hypertension with a mPAP above 25 mmHg, moderate PH with a mPAP above 35 mmHg, and severe PH with a mPAP > 50 mmHg. The liver transplantation-related mortality in the group with severe and moderate PH was 100 and 50 percent, respectively, while no mortality was reported among patients with a mild form of PoPH. Therefore, severe PoPH was found to be an absolute contraindication to elective transjugular intrahepatic portosystemic shunt. At least a moderate PoPH is a relative contraindication for LT due to its high postoperative mortality [9].

The majority of PAH therapy trials have not included PoPH patients or have underrepresented them; nevertheless, therapy for pulmonary hypertension in patients with established PoPH should be conducted according to PAH therapy guidelines, and clinicians should follow the general treatment principles of PAH. So far, Savale et al. have presented an analysis of the PAH-targeted treatment of the largest group of patients with PoPH who were included in the French registry [10]. The majority of patients (74%) in this registry were initially on monotherapy, more often with a phosphodiesterase-5 inhibitor (71%) than with an endothelin-receptor antagonist (27%). A substantial improvement was also noted in the functional class (*p* < 0.001), 6 MWD (*p* < 0.0001), and PVR (*p* < 0.0001) [10]. A small group of patients with PoPH was treated with riociguat as part of the PATENT study. The guanylate cyclase stimulator was well tolerated in patients with PAH and PoPH. It also led to improvements in their exercise and functional capacity [11]. PORTICO has been the only randomized controlled trial, which was dedicated to the treatment of PoPH and randomized 85 patients to receive macitentan treatment or a placebo [12]. Macitentan was well tolerated and significantly improved PVR; however, there were no differences in the secondary outcome measures which included the following: the functional class, 6 MWD, and NT-proBNP [12]. It is challenging for clinicians to use PAH-specific therapies because of their hepatic metabolism, and the symptoms of liver disease tend to overlap with PAH-specific side effects [13].

Patients with mild pulmonary hypertension, i.e., a mPAP below 35 mmHg, do not appear to benefit from targeted treatment for pulmonary arterioles, but such treatment should be considered in patients with a mPAP above 35 mmHg. Prior to LT, PoPH therapy should lower the pressure below 35 mmHg and lower the resistance below five Wood units [6].

There is a strong correlation between the severity of liver disease and the survival of patients with PoPH [10]. Among patients with mild liver disease, pulmonary hypertension progression is the leading cause of death [5]. PoPH patients have poorer survival and all-cause hospitalization rates compared to those with IPAH [14]. Long-term survival is highest among liver transplant patients [5].

Hypoxemia and dilated intrapulmonary arteries are characteristic of hepatopulmonary syndrome (HPS) [5]. The most common cause of HPS is portal hypertension and cirrhosis [6]. It may also occur as a consequence of acute and chronic hepatitis, acute liver failure, and vascular abnormalities that restrict hepatic venous outflow to the lungs (cavopulmonary shunts) [6]. According to the European Respiratory Society guidelines, the severity of hepatopulmonary syndrome (HPS) is classified as follows: mild (PaO_2_ ≥ 80 mmHg), moderate (PaO_2_ 60–79 mmHg), severe (PaO_2_ 50–59 mmHg), and very severe (PaO_2_ < 50 mmHg) [6]. The presence of HPS significantly worsens patients’ prognosis. The mortality and morbidity rates of patients with HPS are doubled and appear to be the highest in those who develop severe hypoxemia (PaO2 < 50 mmHg) [6]. Therefore, HPS screening should be considered in LT candidates [6]. Aside from oxygen administration and maintaining arterial blood oxygen saturation above 88%, there is no specific treatment for this syndrome. Patients with HPS should be favoured on LT lists and transplanted before they develop severe hypoxemia, because the syndrome usually improves after LT [15]. Moreover, HPS and PoPH may occur concomitantly in patients with portal hypertension [5]; consequently, echocardiography often reveals the coexistence of HPS and PoPH. Contrast-enhanced transthoracic echocardiography and lung perfusion scans are useful methods for detecting intrapulmonary vascular dilatations [16]. Contrast echocardiography results are considered to be positive in patients who show a delayed appearance of contrast in the left heart chambers [17]. It is diagnosed as subclinical HPS when there is evidence of intrapulmonary vasodilation on echocardiography without hypoxemia.

We conducted this study in order to evaluate the occurrence of the echocardiographic features of PoPH and HPS in candidates for LT. We hypothesized that our analysis would allow us to determine the frequency with which liver transplantation candidates require further invasive diagnostics for pulmonary hypertension at specialized centers, as well as the frequency with which they may need targeted pulmonary arteriole treatment as a bridge to transplantation to improve their prognosis. Additionally, this study includes a comprehensive review of the current literature.

### Summary of the Introduction

The prevalence of PoPH is relatively low; however, its presence significantly worsens patients’ prognosis. When diagnosed, PoPH can be effectively treated, and specific therapies can lead to a substantial reduction in pulmonary circulation pressure, facilitating the safe performance of liver transplantation. The mortality and morbidity rate of patients with HPS are doubled, which appears to be the highest in those who develop severe hypoxemia [6]. Therefore, HPS screening should be considered in LT candidates [6]. Patients with HPS should be favoured on the LT list and transplanted before they develop severe hypoxemia, because the syndrome usually improves after LT [15]. Screening including an annual electrocardiogram, echocardiography, and BNP testing should be considered for candidates for transplantation even in the absence of symptoms. Echocardiography is recommended as a first-line method for the non-invasive diagnosis of pulmonary hypertension and serves as a valuable screening tool for patients being evaluated for LT. As a result of the high mortality rate in pulmonary hypertension patients undergoing LT, even patients with an intermediate probability of it should be referred to RHC. The objective of this study was to thoroughly assess the occurrence of echocardiographic signs indicative of PoPH and HPS in candidates for LT.

## 2. Material and Methods

This was a retrospective, single-centre analysis of the echocardiographic parameters of candidates for liver transplantation. All liver transplantation candidates underwent standardized transthoracic echocardiography, conducted by an experienced cardiologist in accordance with the latest European Association of Cardiovascular Imaging guidelines [18]. Examinations were conducted in the left lateral position. Right (RV) and left ventricular (LV) dimensions were measured in the parasternal long-axis and apical four-chamber views at the level of the mitral and tricuspid valve tips during late diastole, as indicated by the R wave on the continuous electrocardiogram. Tricuspid regurgitation (TR) was qualitatively assessed using color Doppler imaging, and the tricuspid regurgitation peak gradient (TRPG) was calculated using the simplified Bernoulli formula based on the peak velocity of tricuspid regurgitation flow [19]. A measurement of the inferior vena cava and an assessment of its collapsibility completed the examination. The right ventricular systolic pressure (RVSP) was determined using the Bernoulli equation, based on the peak velocity of tricuspid regurgitation and an estimated right atrial pressure. Interventricular septal flattening was qualitatively assessed, and the pulmonary ejection acceleration time (AcT) was measured within the right ventricular outflow tract, just below the pulmonary valve. In the M-mode presentation, tricuspid annular peak systolic excursion was used to assess the right ventricular function. By using the apical four-chamber and two-chamber views of the left ventricle during systole and diastole, Simpson’s formula was used to calculate the left ventricular ejection fraction (LV EF). A current recommendation was followed for evaluating the valvular morphology and function [20]. Digital recordings of the examinations were kept and reviewed as needed.

The echocardiographic probability of pulmonary hypertension (PH) was classified as follows. A low probability was assigned to patients with a tricuspid regurgitation peak gradient (TRPG) < 31 mmHg and no other echocardiographic signs indicative of PH. An intermediate probability was noted in individuals with a TRPG < 31 mmHg and at least two of the following criteria: A RV/LV diameter ratio > 1.0; a flattened interventricular septum; a pulmonary ejection acceleration time < 105 ms; a mid-systolic “notch”; an early diastolic pulmonary regurgitation velocity > 2.2 m/s; a distended inferior vena cava > 21 mm with reduced inspiratory collapse; a right atrial area (end-systole) > 18 cm^2^; a or pulmonary artery diameter > 25 mm. Additionally, patients with a TRPG ranging from 32 to 46 mmHg without these signs were also categorized as having an intermediate probability. A high probability of PH was diagnosed in this group if additional signs were present, and a high probability was specifically defined as a TRPG > 46 mmHg [1,2].

Contrast echocardiography was conducted by agitating 10 mL of saline in two 10 mL syringes connected via a three-way stopcock, resulting in the formation of microbubbles. The agitated saline was then injected through an intravenous line in the right upper extremity. To visualize the microbubbles, a 5 MHz phased array transducer was used to obtain the parasternal four-chamber view. The presence of intracardiac shunting, such as that from a persistent foramen ovale or atrial septal defect, is indicated by the early appearance of microbubbles in the left heart within one to two cardiac cycles following their detection in the right heart chambers [17]. Conversely, a delayed visualization of microbubbles (after three cardiac cycles) in the left heart chambers suggests intrapulmonary right-to-left shunting; this was considered a positive test result if observed in any of the three injections. A negative test result was defined as the absence of contrast in the left heart chambers following all three injections [21].

### Statistical Analysis

Data that exhibited a normal distribution are presented as means accompanied by standard deviations, while data lacking a normal distribution are presented as medians with ranges. Qualitative variables were compared using the chi-square test. The Mann–Whitney test was employed to analyze data that did not follow a normal distribution, whereas normally distributed data were analyzed using Student’s *t*-test. All statistical analyses were conducted using STATISTICA data analysis software (StatSoft, Inc., Tulsa, OK, USA, 2011, version 10, www.statsoft.com (accessed on 7 July 2024)). A *p*-value of less than 0.05 was deemed statistically significant.

## 3. Results

In total, 152 LT candidates (67 F, age 50.6 year) were included in the analysis. Liver failure was most often caused by hepatitis C (n = 46, 30%) and B (n = 15, 10%). In our study, 24% (n = 37) of patients were diagnosed with alcoholic liver disease. Cholangitis (n = 22, 14.5%) or autoimmune hepatitis (n = 9, 6%) occurred less frequently. Most patients (88%) were in Child–Pugh stage B or worse, and the mean MELD score was 12.67 at the time of a cardiological assessment before liver transplantation (Table 1).

Only one patient had an LVEF reduced to 45%. The mean LVEF was 64.4% since every fifth LT candidate had a described hyperkinetic contractility and an LVEF above 65%. The hyperkinetic contractility of the left ventricle resulted in accelerated flow through the aortic valve, and the average velocity was 1.43 m/s; additionally, six (4%) of the total patients were diagnosed with aortic stenosis. Mild mitral stenosis coexisted with moderate aortic stenosis in one case. The mean pulmonary velocity reached 1.01 m/s; however, 37 patients (24%) had a velocity over 1 m/s, which was most likely due to a right hyperkinetic ventricle. The average tricuspid annular plane systolic excursion was 26 mm, but 36 candidates for LT had one more than 29 mm.

The estimated echocardiographic probability of PH was high in one patient. In 15 cases, the criteria for the intermediate probability of PH were met. The vast majority of patients (n = 136, 89%) presented a low echocardiographic probability of PH, and according to the ESC guidelines, they did not require further diagnostics for PoPH (Table 2). The additional echocardiographic signs suggestive of pulmonary hypertension have been provided in Table 3.

Only a 57-year-old woman with a body mass index of 22.8 kg/m^2^ was found to have a high echocardiographic probability of PH, whose liver failure was caused by primary cholangitis (autoimmune) and hepatitis B. In addition to a high TRPG (tricuspid regurgitation velocity 4.3 m/s), echocardiography revealed other signs that increase the risk of PoPH: the shortening of the pulmonary ejection AcT with the notching of the ejection spectrum, the enlargement of the right atrium area (19.5 cm^2^), and the slight flattening of the interventricular septum. A contrast examination revealed the presence of few contrast bubbles in the cavities of the left heart after four to five cycles. Within 3 months, a computed tomography scan of the coronary arteries was performed, but no abnormalities were found. The patient was scheduled for right heart catheterization, but she rapidly deteriorated. She was admitted to the Transplantation Surgery Department in a serious condition with a MELD score of 19 and consequently died. The fatal outcome seems to confirm that pulmonary hypertension significantly worsens LT candidates’ survival.

Contrast echocardiogram was performed in 99 patients. In 32 cases, the contrast echocardiogram results (32 of 99) were negative, and the remaining 63 patients had the visualization of microbubbles in the left heart chambers after an average of six (between three and nine) contractions after administration. The presence of intracardiac shunting was suggested in four cases, as indicated by the early appearance of microbubbles in the left heart within one to two cardiac cycles after their detection in the right heart. Among 29 patients with a positive contrast echocardiogram, only a few microbubbles were observed. Multiple microbubbles were found in 38 LT candidates. Only three patients were diagnosed with HPS, and three more were recommended for further testing.

## 4. Discussion

One to two percent of patients with liver disease and portal hypertension develop pulmonary arterial hypertension [5]. According to Kouvul et al. PoPH is seen in 5–8% of liver transplantation candidates [22]. Our study only included transplant candidates, and only one patient had a high probability of having pulmonary hypertension based on echocardiography. If the diagnosis of PoPH had been established by the catheterization of the right heart, the incidence of pulmonary hypertension in our group would have been only 0.6%, which is much lower than in the sources cited above. It is worth considering whether the characteristics of our study group were different. PoPH patients (n = 637) enrolled in the French registry had an average age of 55 years (mean age 55 ± 10 years) and were slightly more often male (58%) [4]. Fifty-seven percent of the patients had mild cirrhosis, classified as Child–Pugh stage A, with a median MELD score of 11 [4]. In the REHAP registry, 237 patients with PoPH (mean age 53.9 ± 10.3 years; 55.3% male) were eligible for analysis [3]. Viral hepatitis was the most frequent underlying liver disease (45%) and alcoholic liver disease was established in 35% of patients with PoPH in the Spanish registry. Unfortunately, no information is available regarding Child–Pugh stage in the REHAP registry [3]. The REVEAL registry included 174 patients with PoPH [2]; the mean age was 53 ± 10 years and 52% were female. The type and severity of liver disease are not available in the REVEAL registry [2]. The patients included in our analysis were slightly younger (mean age 50.6 years), with a similar percentage of them being male (56%). As in the Spanish REHAP registry, in our study, liver failure was also mainly caused by viral hepatitis B + C (40%) and alcoholic liver disease (25%). In our analysis, the vast majority of patients (88%) had moderate liver failure with Child–Pugh stage B; the median MELD score was 12.7. However, the extent of underlying liver dysfunction or the hepatic venous pressure gradient did not appear to be correlated with portopulmonary hypertension (PoPH).

The risk of portopulmonary hypertension increases with female sex, autoimmune liver diseases, elevated estradiol levels, and splenectomy [13]. This confirms our observation, because in our study, the highest probability of PH was held by a woman with autoimmune liver failure. However, in our group, the male sex was slightly more common, which reduced the risk of developing PoPH, but autoimmune factors leading to the development of liver disease occurred in every fifth patient. Hormonal and immunologic factors may affect the development of PoPH [23]. Interestingly, Kawut et al. demonstrated that hepatitis C infection is associated with a decreased risk of developing PoPH [23], which accounted for 30% of our patients’ liver failures.

In accordance with the ESC’s recommendations, the diagnostic approach for PoPH should be the same as for pulmonary arterial hypertension. However, echocardiographic screening for pulmonary hypertension, regardless of the occurrence of symptoms, is recommended only in patients qualified for LT [2,3]. It has been found that a transthoracic echocardiogram can detect PoPH with an 85% sensitivity and 83% specificity. Echocardiogram is a non-invasive tool that can be performed at the patient’s bedside and estimates the pressure in the pulmonary circulation. However, imaging conditions may be limited by ascites and the enlargement of the liver. As patients with a liver disease often have an elevated cardiac output, the tricuspid regurgitation velocity tends to overestimate the pulmonary artery pressure in these patients [24,25]. Consequently, the researchers propose different cut-off points for the TRPG that should be used to determine if patients need further diagnostic testing. There is an ongoing debate regarding whether the cut-off point for estimated right ventricular systolic pressure (eRVSP) should differ for liver transplantation candidates. The International Liver Transplant Society recommends conducting right heart catheterization (RHC) in patients with an eRVSP exceeding 50 mmHg and/or those exhibiting signs of right ventricular dysfunction or hypertrophy [6]. In order to confirm the presence of PoPH, Korbitz recommends RHC when RVSP estimates are over 45 mmHg [7]. Only one patient in our group met the criteria for a high echocardiographic probability of PH; unfortunately, the disease led to death before the patient had RHC performed.

Echocardiography does not differentiate precapillary and postcapillary PH, which supports the need for invasive RHC and is useful for determining the type of pulmonary hypertension [1,5]. There is a possibility that pulmonary hypertension may develop in LT candidates with valvular left heart disease [20]. The targeted treatment of pulmonary arterioles is recommended only in the case of confirmed pulmonary arterial hypertension [3]. PAH therapy can effectively reduce pulmonary circulation pressure and the risk of peri-transplantation complications in PoPH patients [25,26].

It is always advisable to consider hepatopulmonary syndrome as a differential diagnosis when hypoxemic patients have underlying liver disease, especially since patients with HPS are often mildly symptomatic or asymptomatic [27]. Clinical signs may include dyspnea on exertion or at rest, digital clubbing, and cyanosis [6]. A fourth of HPS patients suffer from platypnea, which occurs when dyspnea worsens when they move from a supine position to an upright position, as well as orthodeoxia, which can be considered if the decrease in SpO2 reaches 5% or more when the patient moves from a supine to an upright position. Pulse Oximetry seems to be an optimal screening test for detecting HPS [27].

There is a prevalence of HPS between 5% and 30% [3] among LT candidates, and it is most commonly seen in patients with cirrhosis and portal hypertension. HPS is more frequent than PoPH; therefore, the signs of right-to-left shunts are also more prevalent in echocardiography [27]. The gold standard of the diagnostic approach is a contrast-enhanced echocardiography. If microbubbles are seen in the left heart after three or more cardiac cycles after intravenous injection, they indicate dilated intrapulmonary arteries or shunts from the right heart. An alternative method to reveal intrapulmonary vascular dilatations is a 99 mTechnetium-labeled macro aggregated albumin lung perfusion scan. In our study, the transthoracic echocardiogram of every third patient showed microbubbles in the left heart chamber after three to nine cycles, but only few patients were diagnosed. HPS is frequently under-diagnosed, which is confirmed by the analysis of researchers from Phoenix’s University of Arizona College of Medicine, who showed that, of 42,749 patients with cirrhosis, only 194 cases (0.45%) were diagnosed with HPS, in addition to only half of them having delayed shunting [28].

Screening for HPS may be considered in LT candidates due to the fact that the presence of HPS, as well as PH, worsens their prognosis and quality of life [29]. Furthermore, the more advanced the HPS is, and the more severe the hypoxemia is and the higher the mortality rate is. LT candidates should undergo HPS testing (pulse oximetry, contrast echocardiography, and lung perfusion scanning) and LT should be performed before severe HPS develops [6]. Liver transplantation is the sole therapeutic option for hepatopulmonary syndrome and leads to a complete resolution of the condition. Long-term survival in patients with HPS significantly improves following liver transplantation, effectively serving as a cure for the disease [6].

### Limitations of the Study

The impact of this study is constrained by the small sample size, despite Poland being a country with a population of 38 million, where 330 and 335 liver transplants were performed in 2019 and 2022, respectively. Our study included data from 152 liver transplantation candidates, which represents nearly half of the annual liver transplants performed in Poland [30]. Notably, only one patient in the study group (one out of one hundred and fifty-two) was found to have a high echocardiographic probability of pulmonary hypertension (PH) and required further invasive diagnostics. This patient was classified as high-risk; however, the diagnosis of PH could not be confirmed due to their rapid clinical deterioration. Even if right heart catheterization (RHC) had been performed and portopulmonary hypertension (PoPH) confirmed, it would have highlighted the rarity of PoPH among transplantation candidates. Conversely, echocardiography is a non-invasive, cost-effective, and widely accessible diagnostic tool. Therefore, it is advisable to utilize echocardiography in liver transplantation candidates to facilitate their diagnosis and treatment, potentially benefiting at least one in one hundred and fifty-two patients.

## 5. Conclusions

Our analysis indicates that features of portopulmonary hypertension (PoPH) and a high probability of pulmonary hypertension (PH) are quite rare among liver transplantation candidates, while echocardiographic signs indicative of hepatopulmonary syndrome (HPS) are more commonly observed. Therefore, it is essential to screen liver transplant candidates for both PoPH and HPS, as both conditions significantly deteriorate their prognosis. As noted, both hepatopulmonary syndrome (HPS) and pulmonary hypertension (PH) adversely affect prognosis and quality of life in patients. Therefore, future studies are warranted to assess whether specific PH treatments could improve survival outcomes in PoPH patients awaiting transplantation.

## Figures and Tables

**Table 1:** Study group characteristics.

	All patients n = 152
Female/Male, n	67/85
Age, years	50.61 (19–66)
BMI, (kg/m^2^)	25.78 (±6.66)
MELD score	12.67 (6–31)
Serum bilirubin (mg/dL)	3.5 (0.28–29.9)
Serum creatinine (mg/dL)	0.98 (0.4–9.95)
INR	1.5 (0.9–2.5)
Serum albumin (g/dL)	2.9 (0.2–5)
Child–Pugh score ≥ 7	n = 82/n = 93
Hepatitis C	n = 46 (30%)
Alcoholic liver disease	n = 37 (24%)
Cholangitis	n = 22 (14.5%)
Hepatitis B	n = 15 (10%)
Autoimmune hepatitis	n = 9 (6%)

INR—international normalized ratio.

**Table 2:** Echocardiographic characteristics of the study group.

Peak Tricuspid Regurgitation Velocity	n = 152	Additional Echocardiographic Signs Suggestive of PH	Echocardiographic Probability of PH
>3.4 m/s	n = 1	n = 1	High: n = 1
2.9–3.4 m/s	n = 12	n = 0	High: n = 0 Intermediate: n = 12
<2.8 m/s	n = 120	n = 3	Intermediate: n = 3 Low: n = 117
Unmeasurable	n = 19	n = 0	Low: n = 19

PH—pulmonary hypertension.

**Table 3:** Additional echocardiographic signs suggestive of pulmonary hypertension.

Ventricles	Pulmonary Artery	Inferior Vena Cava and Right Atrium
Right-to-left ventricle basal diameter/area ratio > 1.0	Right ventricular outflow tract acceleration time < 105 ms and/or mid-systolic notching	Inferior vena cava diameter > 21 mm with decreased inspiratory collapse (<50% with sniff or <20% with quiet inspiration)
Flattening of the interventricular septum (left ventricle eccentricity index > 1.1 in systole and/or diastole)	Early diastolic pulmonary regurgitation velocity > 2.2 m/s	Right atrium area (end-systole) > 18 cm^2^
Tricuspid annular plane systolic excursion to systolic pulmonary arterial pressure ratio < 0.55 mm/mmHg	Pulmonary artery diameter > aortic root diameter Pulmonary artery diameter > 25 mm	

## Data Availability

The original contributions presented in the study are included in the article, further inquiries can be directed to the corresponding author.
